# Mathematical Model of Bursting in Dissociated Purkinje Neurons

**DOI:** 10.1371/journal.pone.0068765

**Published:** 2013-08-13

**Authors:** Michael D. Forrest

**Affiliations:** Department of Computer Science, University of Warwick, Coventry, West Midlands, United Kingdom; Georgia State University, United States of America

## Abstract

*In vitro*, Purkinje cell behaviour is sometimes studied in a dissociated soma preparation in which the dendritic projection has been cleaved. A fraction of these dissociated somas spontaneously burst. The mechanism of this bursting is incompletely understood. We have constructed a biophysical Purkinje soma model, guided and constrained by experimental reports in the literature, that can replicate the somatically driven bursting pattern and which hypothesises Persistent Na^+^ current (I_NaP_) to be its burst initiator and SK K^+^ current (I_SK_) to be its burst terminator.

## Introduction

*In vitro*, without any synaptic input, Purkinje neurons can spontaneously fire action potentials in a repeating trimodal pattern that consists of tonic spiking, bursting and quiescence [1]–[6]. So, Purkinje cells have been observed to burst, as a component of the trimodal firing pattern. This bursting has been shown to be dendritically driven, by dendritic Ca^2+^ spikes [3], and has been modeled [6]. Purkinje cells also burst in response to Climbing fiber (CF) input; a CF input produces a Ca^2+^ spike in the dendrites and this propagates to, and produces a burst event (“complex spike”) at, the soma [7]–[10].

*In vitro*, Purkinje cell behaviour is sometimes studied in a dissociated soma preparation in which the dendritic projection has been cleaved. A fraction of these dissociated somas are quiescent whilst others spontaneously fire simple spikes [11] or spontaneously burst [12]. Given that this preparation has no dendrites, the drive to its bursting must be of somatic origin. Hence, this bursting must be distinct from the bursting in the trimodal pattern of firing, which has been shown to be dendritically driven [3], [6]. Furthermore, it must be distinct from burst events in response to CF input, which have been shown to be dendritically driven [7]–[10]. Indeed, the bursting waveform of isolated Purkinje somata is distinct from the *stereotypical* waveform of dendritically driven bursts.

Dendritically driven bursts have an ongoing increase in firing rate and termination by a more rapid increase in firing rate, with a decrease in spike height. The burst rides upon a slow wave of depolarisation and ends with a rapid depolarization. This depolarization is followed by a rapid hyperpolarization that persists through the interburst interval [3], [6]. By contrast, somatically driven bursts are without any systematic change in firing rate or spike height upon burst progression [12]. The burst's final spike height is not dramatically smaller and the burst does not ride upon a wave of depolarisation. In addition, somatically driven bursts tend to be shorter (two to four spikes per burst) than dendritically driven bursts (which can have hundreds of spikes per burst, although most typically have under ten).

The mechanism of the isolated soma's spontaneous bursting is incompletely understood. Swensen and Bean [12] studied bursts in this experimental preparation; but they parsed the ionic basis to an imposed, elicited burst produced upon a depolarising current injection. Hence, they did not focus upon spontaneous bursting; so, to what extent are their findings applicable to spontaneous bursting? Here we have constructed a biophysical Purkinje soma model, guided and constrained by the experimental findings of Swensen and Bean [12], which can replicate the somatically driven spontaneous, bursting pattern. This model provides an ionic basis to spontaneous bursting, in a dissociated Purkinje soma. It hypothesises Persistent Na^+^ current (I_NaP_) to be the burst initiator and SK K^+^ current (I_SK_) to be the burst terminator. Swensen and Bean [12] implicate the importance of an inward TTX-sensitive (Na^+^) current and we go further in resolving this as I_NaP_. We find that Fast Na^+^ (I_NaF_) or Resurgent Na^+^ (I_NaR_) cannot generate bursts.

Why do some dissociated Purkinje somas spontaneously fire simple spikes [11] rather than spontaneously burst [12]? Our model provides an explanation; higher BK and/or SK activation (which can be driven by greater P-type Ca^2+^ current flow, and elevated [Ca^2+^]_i_) switches a dissociated Purkinje soma from bursting to tonic spiking. So, the bursting mode is “gated” by the BK and SK currents, which are in turn “gated” by the P-type Ca^2+^ current (I_CaP_).

An isolated Purkinje cell soma is a severely reduced system. Is its bursting mode an artefact of the isolated soma system? Or can it occur in the full Purkinje cell morphology? If so, does it occur *in vivo* and does it have a physiological role? We do not address the latter issues here. But we do use the model to propose that the full Purkinje cell morphology *can* express the same bursting form as that observed in isolated Purkinje somas. *In vitro*, if the P-type voltage-gated Ca^2+^ current (I_CaP_) is pharmacologically blocked in a full Purkinje cell morphology, its inherent tonic or trimodal firing pattern can be replaced with a phase of bursting and then a depolarisation block [1], [2]. The waveform of this bursting is without the stereotypical change in firing rate/spike height, upon burst progression, that characterises dendritically generated bursting [3]. In fact, it is reported as similar to that observed in mechanically dissociated somas [3]. So, we suggest that this bursting is somatically driven. Our soma model can replicate this transition upon a P-type Ca^2+^ channel block: tonic firing to bursting (somatically driven waveform) and finally to depolarisation block.

We propose that cerebellar Purkinje cells have two distinct bursting modes – dendritically driven and somatically driven. The somatically driven bursting mode is observed in dissociated Purkinje somas and we use our model to propose that it can also be observed in full Purkinje cell morphologies, all be it in an artificial system – in cerebellar slices with the perfusion of a drug. We propose the possibility that Purkinje cells express a somatically driven bursting form under physiological conditions and utilise it in information coding.

## Materials and Methods

Numerical *s*imulations were performed with the NEURON 5.6 simulator [13], using its backward Euler integration method and 25 µs time steps. The model soma is a single cylindrical compartment with a length and diameter of 22 µm; its specific membrane capacitance (*C*_m_) is 0.8 µF/cm^2^ [14]. The soma has highly TEA sensitive (I_K_fast_), moderately TEA sensitive (I_K_mid_) and TEA insensitive (I_K_slow_) voltage-gated K^+^ currents, a BK voltage-and-Ca^2+^-gated K^+^ current (I_BK_), a P-type Ca^2+^ current (I_CaP_), a hyperpolarization activated cation current (I_H_), a leak current (I_L_) and an intracellular Ca^2+^ dynamics abstraction - all sourced from Khaliq et al. [11]. In addition, it has a Resurgent Na^+^ current (I_NaR_) (description from [15]), a T-type Ca^2+^ current (I_CaT_) and a Fast Na^+^ current (I_NaF_) (descriptions from [16]). The model currents have equations and kinetic parameters as described in their source literature, but with the modification of current density values to those shown in Table 1. This isolated somatic compartment spontaneously fires in a simple spiking form, as many isolated Purkinje somata do. It can be switched to bursting (4 spikes per burst) by adding a Persistent Na^+^ current (I_NaP_, density = 4 mS/cm^2^; description from [17]) and a SK Ca^2+^-gated K^+^ current (I_SK_, density = 4 mS/cm^2^; description from [18]).

**Table 1:** Maximal current conductances in the model soma.

Current	Density (mS/cm^2^)
Resurgent Na^+^	156
Fast Na^+^	0.1
P-type Ca^2+^	0.52
T-type Ca^2+^	0.1
BK K^+^	72.8
Highly TEA sensitive K^+^	41.6
Moderately TEA sensitive K^+^	20.8
TEA insensitive K^+^	41.6
I_H_	1.04
Leak	0.52
Persistent Na^+^	4
SK K^+^	4

Without the inclusion of the two currents highlighted in grey (Persistent Na^+^ current and SK K^+^ current), the model soma fires tonic spikes. With their inclusion, at the densities presented, the model soma bursts.

All model parameters were established by prior literature (as referenced) except the current densities, which are ill constrained by published experimental data. These variables were tuned manually [19] by iteratively running the model with different current density values and observing which combination of these gave the best fit between real and model Purkinje soma output. We tuned the model manually because, drawing from our experience, we reasoned that with the reasonable number of parameters, and with the significant complexity/timeframe of the model behaviours sought, parameter optimization algorithms would struggle to converge upon a good solution. Principally, we tuned the model to replicate the characteristics of bursting in an isolated Purkinje soma [12].

### Model equations

*C_m_* is the membrane capacitance, *I* is the current, *V* is the membrane potential in mV as a dimensionless quantity, *t* is time, E_K_ is the reversal potential for K^+^ (−88 mV), E_Na_ is the reversal potential for Na^+^ (+60 mV), E_L_ is the reversal potential for the Leak current (−60 mV), E_h_ is the reversal potential for the hyperpolarisation activated cation current (−30 mV), *T* is temperature 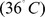 and g_max_ is the maximal conductance (“current density”).

g_max_ values, for the different currents, are shown in Table 1.

*m*, *h* and *z* are Hodgkin-Huxley “particles”/gates [**30**]; for example, for the *m* Hodgkin-Huxley gate:
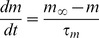
(1)The voltage (and/or intracellular calcium) dependence of a Hodgkin-Huxley (H-H) current [**30**] can be expressed by stating, for each H-H gate (e.g. for the *m* gate), either [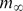,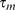] OR [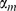,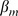]. These entities are voltage (and/or intracellular calcium) dependent. The latter set can give the former set through the relations:
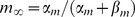
(2)

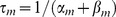
(3)


#### Master equation



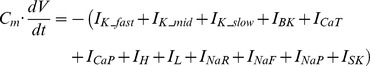
(4)If I_NaP_ and I_SK_ are removed, then the model is switched from bursting to tonic spiking.

#### Highly TEA sensitive K+ current [11]



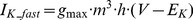
(5)

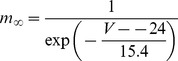
(6)

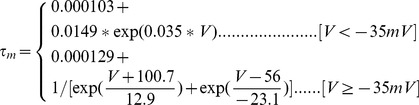
(7)

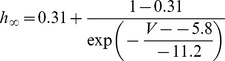
(8)

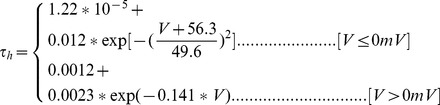
(9)

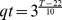
; *T* is temperature in degrees centigrade (36). The time constants 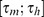 are divided by *qt*, in a modification to the original description [**11**], to account for temperature.

#### Moderately TEA sensitive K+ current [11]



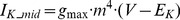
(10)

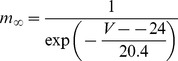
(11)

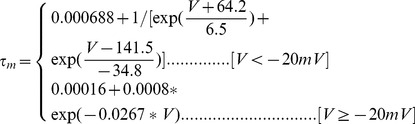
(12)

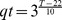
; *T* is temperature in degrees centigrade (36). The time constant 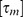 is divided by *qt*, in a modification to the original description [**11**], to account for temperature.

#### TEA insensitive K+ current [11]



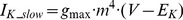
(13)

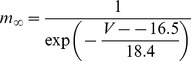
(14)

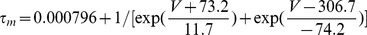
(15)

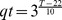
; *T* is temperature in degrees centigrade (36). The time constant 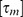 is divided by *qt*, in a modification to the original description [**11**], to account for temperature.

#### P-type Ca2+ current [11]



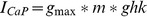
(16)Goldman-Hodgkin-Katz (ghk) equation:
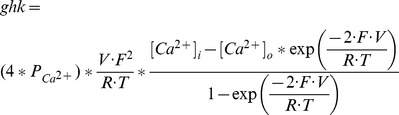
(17)P_Ca_^2+^ is 5*10^−5^ cm/sec, [Ca^2+^]_i_ = 100 nM, [Ca^2+^]_o_ = 2 mM, T = 295 K, F is the Faraday constant and R is the gas constant. [Ca^2+^]_i_ and [Ca^2+^]_o_ are fixed constants, as seen by this equation – it does *not* access the changing value of [Ca^2+^]_i_ as set by the intracellular Ca^2+^ equations (given later).
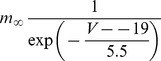
(18)

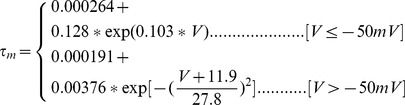
(19)

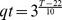
; *T* is temperature in degrees centigrade (36). The time constant 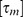 is divided by *qt*, in a modification to the original description [**11**], to account for temperature.

#### Hyperpolarisation activated cation current [11]



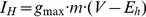
(20)

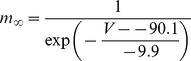
(21)

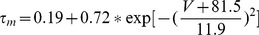
(22)

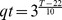
; *T* is temperature in degrees centigrade (36). The time constant 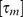 is divided by *qt*, in a modification to the original description [**11**], to account for temperature.

#### BK type K+ current [11]



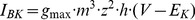
(23)

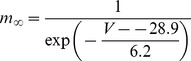
(24)

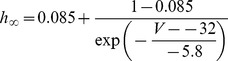
(25)

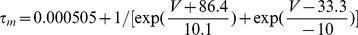
(26)

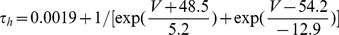
(27)

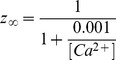
(28)

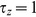
(29)

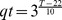
; *T* is temperature in degrees centigrade (36). The time constants 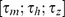 are divided by *qt*, in a modification to the original description [**11**], to account for temperature.

#### Leak current [11]



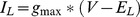
(30)


#### Intracellular Ca2+ concentration [11]

[Ca^2+^] is calculated for the intracellular space within 100 nm of the membrane. [Ca^2+^] changes as I_Ca_^2+^ brings Ca^2+^ into this space and as Ca^2+^ leaves by diffusion to the bulk cytoplasm. The diffusion rate constant, 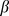, is set to 1/msec.
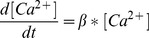
(31)[Ca^2+^] at time step, *t*:
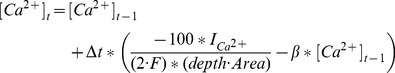
(32)
*F* is the Faraday constant, *depth* = 0.1 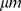 and membrane surface *Area* = 1,521 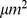. [Ca^2+^] was constrained to not fall below 100 nM by coding of the form:
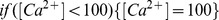



#### Resurgent Na+ current [15]



**Figure 1:**
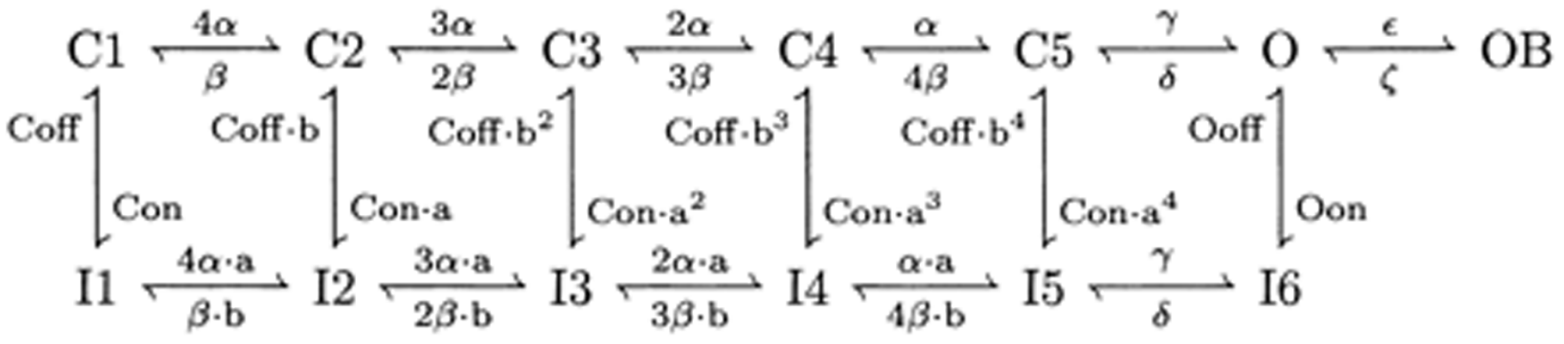
The Resurgent Na^+^ current is described by a Markov scheme [11]. [C1 to C5] denote sequential Closed states; O denotes the Open state. [I1 to I6] denote Inactivated states. OB denotes the state entered by a second mechanism of inactivation, which is hypothesized to be equivalent to Open Channel Block. The rate constants between states are given in Eq. [34], Eq. [35], Eq. [36], Eq. [37], Eq. [38] and Eq. [39].

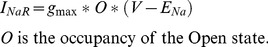
(33)This current is described by a Markov scheme, shown in Figure 1. The rate constants, labelled in Figure 1, are (ms^−1^):
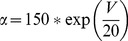
(34)

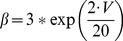
(35)

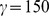
; 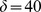; 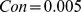; 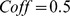; 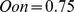; 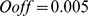


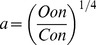
(36)

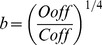
(37)

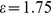
(38)

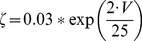
(39)All rate constants are multiplied by 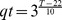; where *T* is temperature in degrees centigrade (36).

#### T-type Ca2+ current [16]



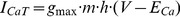
(40)E_Ca_ is +135 mV for this current.
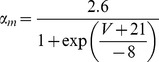
(41)

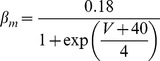
(42)

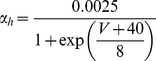
(43)

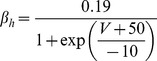
(44)

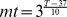
; *T* is temperature in degrees centigrade (36).
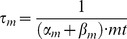
(45)

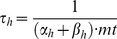
(46)


#### Fast Na+ current [16]



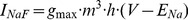
(47)E_Na_ is +45 mV for this current (as opposed to +60 mV).
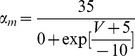
(48)

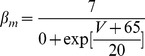
(49)

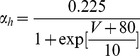
(50)

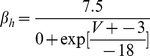
(51)

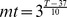
; *T* is temperature in degrees centigrade (36).
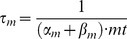
(52)

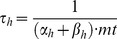
(53)


#### Persistent Na+ current [17]



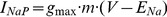
(54)

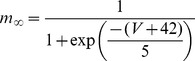
(55)

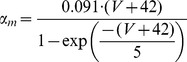
(56)

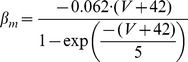
(57)

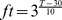
; *T* is temperature in degrees centigrade (36).
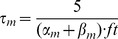
(58)


#### SK type K+ current [18]



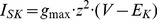
(59)

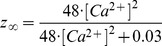
(60)

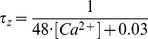
(61)


## Results

### The Purkinje soma model can replicate the somatically driven bursting pattern

**Figure 2:**
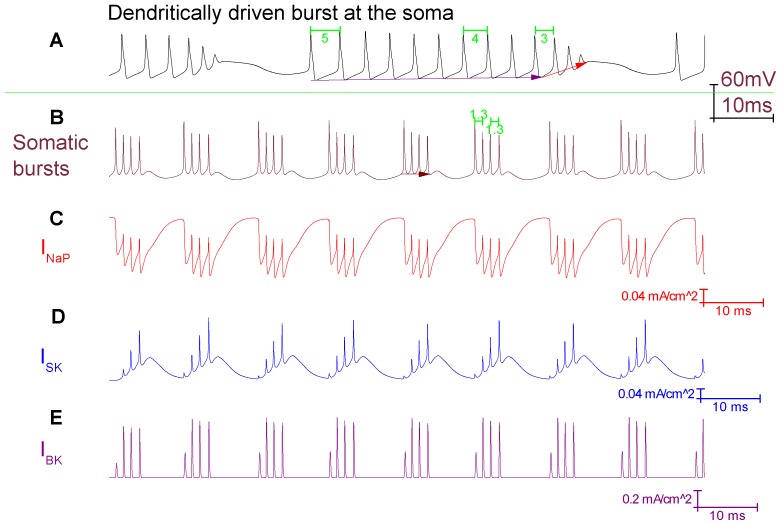
The isolated Purkinje soma model bursts if the I_NaP_ and I_SK_ conductances are both set to 4 mS/cm^2^. These somatically driven bursts have a different waveform than dendritically driven bursts. *A*, Dendritically driven bursts in a full Purkinje cell model (described in [6]) have a very stereotypical waveform with an ongoing increase in firing rate and termination by a more rapid increase in firing rate, with a decrease in spike height. The former is highlighted by the labelled interspike intervals, which shorten upon burst progression. Each burst rides upon a slow wave of depolarisation (highlighted by the gradient of the blue arrow) and ends with a quick depolarization (highlighted by the gradient of the red arrow). It is followed by a rapid hyperpolarization that persists through the interburst interval. *B*, Somatically driven bursts in our isolated Purkinje soma model are, by contrast, without any systematic change in firing rate (refer to the labelled interspike intervals) or spike height upon burst progression and do not ride upon a wave of depolarisation (refer to the flat slope of the brown arrow). In addition, somatically driven bursts tend to be shorter (two to four spikes per burst) than dendritically driven bursts (which can have hundreds of spikes per burst, although more typically have under ten). *C*, I_NaP_ initiates and maintains the bursts in the Purkinje soma model. The Na^+^ current conducted by the somatic I_NaP_ channel (following convention I_NaP_, as an inward current, is represented as negative) is persistent and does not return to baseline after each spike in the burst. This persistence produces the sustained depolarization that initiates and maintains each somatic burst. *D*, I_SK_ is responsible for burst termination in the Purkinje soma model. During a burst, I_SK_ builds in magnitude until it ultimately attains enough strength to terminate it. I_SK_ then resets during the inter-burst interval. *E*, In contrast to I_SK_, I_BK_ (except for an increase at the second spike) declines in magnitude with burst progression and is not responsible for burst termination. *Panel A and B scaling is encoded in the first scale bar (60 mV, 10 ms). Panel C scaling is encoded in the second scale bar (0.04 mA*/cm^2^, *10 ms). Panel D scaling is encoded in the third scale bar (0.04 mA*/cm^2^, *10 ms). Panel E scaling is encoded in the fourth scale bar (0.2 mA*/cm^2^, *10 ms)*.

Figure 2A shows a dendritically driven burst, of the trimodal firing pattern, from the full Purkinje cell model of Forrest et al. [6]. Figure 2B shows a somatically driven burst from our isolated soma model. Dendritically driven bursts have a very stereotypical waveform that contrasts with that of somatically driven bursts. They have an ongoing increase in firing rate and termination by a more rapid increase in firing rate, with a decrease in spike height. The burst rides upon a slow wave of depolarisation and ends with a rapid depolarization. This depolarization is followed by a rapid hyperpolarization that persists through the interburst interval [3], [6]. By contrast, somatically driven bursts are without any systematic change in firing rate or spike height upon burst progression [12]. The burst's final spike height is not dramatically smaller and the burst does not ride upon a wave of depolarisation. In addition, somatically driven bursts tend to be shorter (two to four spikes per burst) than dendritically driven bursts (which can have hundreds of spikes per burst, although most typically have under ten).

### Bursting in the Purkinje soma model: Persistent sodium current is the burst initiator; SK potassium current is the burst terminator

The isolated Purkinje soma model can be switched from spontaneous tonic firing, to spontaneous bursting, by the introduction of the Persistent Na^+^ current (I_NaP_, density = 4 mS/cm^2^) and the SK Ca^2+^-gated K^+^ current (I_SK_, density = 4 mS/cm^2^).

The depolarising Persistent Na^+^ current (I_NaP_) produces the sustained depolarization that initiates and maintains a somatic burst: I_NaP_ is the “burst initiator” (*Figure 2C*). The hyperpolarising Ca^2+^-activated SK potassium current (I_SK_) builds in magnitude with burst progression, until it attains enough strength to terminate the burst, and produce the period of hyperpolarization before the next burst: I_SK_ is the burst “terminator” (*Figure 2D*). So, this bursting can be described as having a [I_NaP_ vs. I_SK_] basis.

### The Purkinje soma model was built with guidance from experimental findings

Swensen and Bean studied bursts in isolated Purkinje cell somas; they parsed the ionic basis to an imposed, elicited burst produced upon a depolarising current injection [12]. Our Purkinje soma model can burst spontaneously, without current injection. We set SK as the model's *spontaneous* burst terminator because Swensen and Bean have shown (experimentally) its importance to *elicited* somatic bursting: “SK increases progressively during bursts and plays an important role in regulating burst duration” [12]; SK is likely the burst terminator, and not BK, because “BK current progressively decreases during the burst, whereas SK current progressively increases” [12]. Our model replicates this rising SK current, and falling BK current, during burst progression (*Figure 2*, *panels D & E*).

We set a Na^+^ current, rather than a Ca^2+^ current, as the depolarising force that initiates and maintains a somatically driven, *spontaneous* Purkinje burst because Na^+^ channel block, by cobalt or TTX, prevents *elicited* bursting [12]. By contrast, Ca^2+^ channel block actually promotes *elicited* burst firing - “Blocking voltage-dependent Ca^2+^ entry by cadmium or replacement of external Ca^2+^ by Mg^2+^ enhanced burst firing” [12]. This promotion is likely manifested by Ca^2+^ channel block decreasing the intracellular Ca^2+^ concentration, which then results in less activation of the hypothesised burst terminator, the Ca^2+^-activated SK channel.

So, by analogy with the experimental findings upon *elicited* somatic bursting, we hypothesise that the depolarising current that initiates and maintains *spontaneous* bursting is likely a Na^+^ current. Manual tuning of Persistent Na^+^ (I_NaP_), Fast Na^+^ (I_NaF_) and Resurgent Na^+^ (I_NaR_) current densities showed only I_NaP_ able to generate spontaneous bursts in the model. With this modelling result, we hypothesise that I_NaP_ is the spontaneous burst initiator in real, dissociated Purkinje somas. I_NaP_ has been shown to be present in cerebellar Purkinje cells [20]–[22].

The hyperpolarisation activated cation current (I_H_) contributes to bursting in a number of neuron types [23], [24]. However, Swensen and Bean do not find it involved in *elicited* bursting, in isolated Purkinje somas [12]. Our modelling corresponds with this as I_H_ block (setting the I_H_ current density to 0) has no consequence upon *spontaneous* model bursting (data not shown).

### Characteristics of bursting in the Purkinje soma model

**Figure 3:**
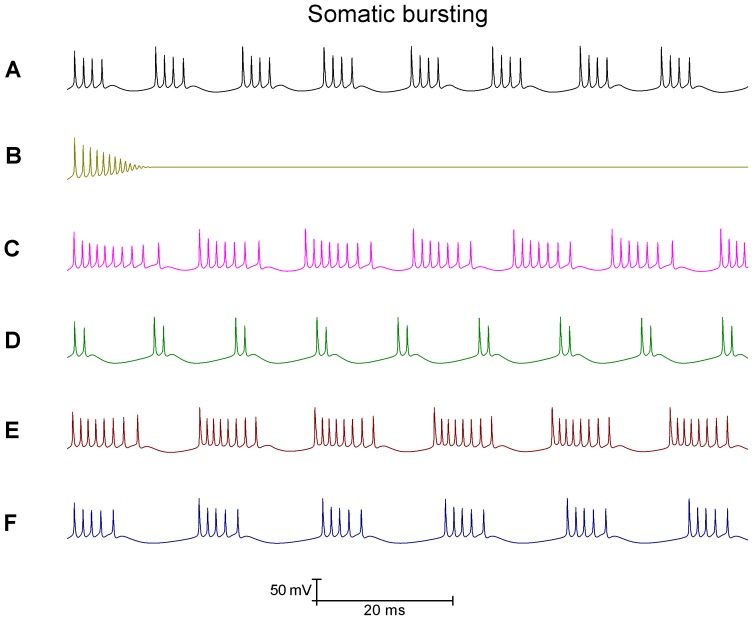
The bursting characteristics of the Purkinje soma model are dependent on a number of parameters. *A*, Bursts in the Purkinje soma model; I_NaP_ and I_SK_ have a current density of 4 mS/cm^2^. *B*, I_NaP_ alone is not sufficient for bursting, I_SK_ is also required. With I_NaP_ present (the burst initiator) and I_SK_ absent (the burst terminator) a burst initiates but cannot terminate. So, the membrane potential becomes “stuck” mid-burst at a depolarised membrane potential. *C*, Increasing the I_NaP_ density (4 to 5 mS/cm^2^) increases the number of spikes per burst (4 to 7). *D*, Increasing the I_SK_ density (4 to 8 mS/cm^2^) decreases the number of spikes per burst (4 to 2). *E*, Increasing the I_NaR_ density (0.156 to 0.3 S/cm^2^) increases the number of spikes per burst (4 to 7). *F*, Increasing the I_CaT_ density (0.1 to 1 mS/cm^2^) increases the number of spikes per burst (4 to 5).

1. I_NaP_ alone is not sufficient for bursting, I_SK_ is also required. If I_NaP_ (4 mS/cm^2^) is introduced, without I_SK_, then the model soma does not burst – it is depolarisation blocked. This is because the introduced I_NaP_ initiates a burst, but there is no I_SK_ to terminate it. So, the soma gets “stuck” mid-burst at a depolarised membrane potential (*Figure 3B*).
2. Increasing the density of the burst initiator, I_NaP_ (from 4 mS/cm^2^ to 5 mS/cm^2^), increases the number of spikes per burst (from 4 to 7) (*Figure 3C*).
3. Increasing the density of the burst terminator, I_SK_ (from 4 mS/cm^2^ to 8 mS/cm^2^), decreases the number of spikes per burst (from 4 to 2) (*Figure 3D*).
4. Increasing the density of Resurgent Na^+^ current (I_NaR_) (from 0.156 S/cm^2^ to 0.3 S/cm^2^) increases the number of spikes per burst (from 4 to 7). I_NaR_ is not the burst initiator and cannot generate bursting *de novo*. However, it can promote I_NaP_ established bursting (*Figure 3E*).
5. Increasing the density of the T-type Ca^2+^ current (I_CaT_) (from 0.1 mS/cm^2^ to 1 mS/cm^2^) increases the number of spikes per burst (from 4 to 5). I_CaT_ is not the burst initiator and cannot generate bursting *de novo*. However, it can promote I_NaP_ established bursting (*Figure 3F*).

### I_BK_ and/or I_SK_ activity can set whether the Purkinje soma model fires tonic spikes or bursts

**Figure 4:**
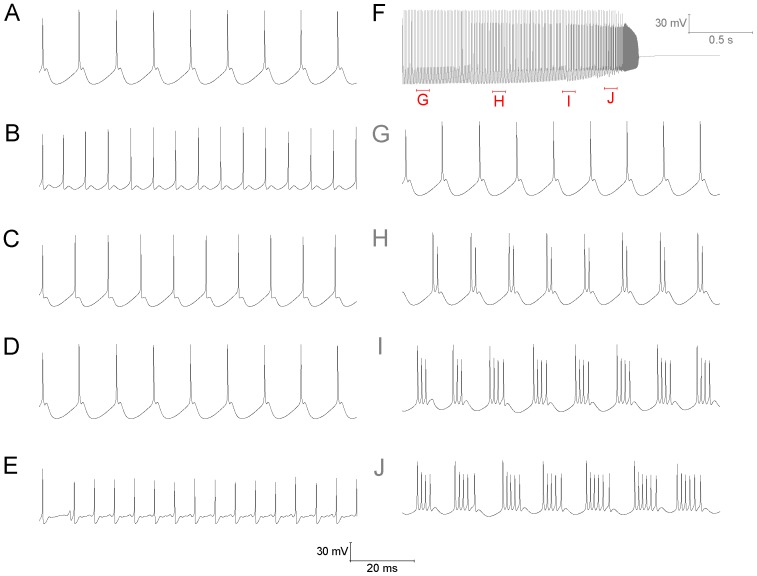
The bursting of the Purkinje soma model is gated by the BK and SK conductances. *A*, Increasing the I_SK_ density (4 to 20 mS/cm^2^) switches the model from bursting to simple spiking. *B*, Increasing the I_BK_ density (0.0728 to 10 S/cm^2^) switches the model from bursting to simple spiking. *C*, Increasing both the SK and BK densities (I_SK_ from 4 to 20 mS/cm^2^. I_BK_ from 0.0728 to 10 S/cm^2^) switches the model from bursting to simple spiking. In this condition bursting is prevented by both I_BK_ and I_SK_ in a redundancy. *D*, Removing I_BK_ does not permit bursting because the I_SK_ block to bursting is still present. *E*, Removing I_SK_ does not permit bursting because the I_BK_ block to bursting is still present. *F*, However, concurrent I_BK_ and I_SK_ removal does permit bursting. For example, decreasing the I_BK_ and I_SK_ densities to zero by an arbitrary function of time (t) [density(t) = density(0)- 0.00001*t] shifts the somatic activity from simple spiking, into bursting and then depolarisation block silence. So, in this panel one can observe the transition from tonic spiking to bursting. *Panels G*, *H*, *I and J* correspond to the labelled parts of *Panel F* and highlight the transition from simple spiking to bursting. *Panel F scaling is encoded in its own scale bar (30 mV, 0.5 s); the scaling of all other panels is encoded in the scale at the bottom of the figure (30 mV, 20 ms)*.

1. Increasing the density of I_SK_ sufficiently (from 4 mS/cm^2^ to 20 mS/cm^2^) can switch the model out of bursting and into simple spiking (*Figure 4A*).
2. Increasing the density of I_BK_ sufficiently (from 0.0728 S/cm^2^ to 10 S/cm^2^) can switch the model out of bursting and into simple spiking (*Figure 4B*).
3. Increasing the density of both I_SK_ and I_BK_ (to 20 mS/cm^2^ and 10 S/cm^2^ respectively) can switch the model out of bursting and into simple spiking (*Figure 4C*). In this condition bursting is prevented by both I_BK_ and I_SK_ in a redundancy. Removing I_BK_ does not permit bursting because the I_SK_ block to bursting is still present (*Figure 4D*). Removing I_SK_ does not permit bursting because the I_BK_ block to bursting is still present (*Figure 4E*).

Bursting occurs when the depolarisation capacity exceeds the repolarisation capacity, and so the potential cannot be repolarised to the resting potential before the onset of the next spike. Raising I_BK_ and/or I_SK_ density, both repolarising entities, can correct this imbalance in the model and set a simple spiking pattern. Conceivably the raising of any repolarising current can act similarly to switch bursting to simple spiking – to gate the bursting/simple spiking duality. However, we specifically hypothesise that real Purkinje somata have gating by both I_BK_ and I_SK_. Hence for bursting to be “unlocked”, both I_BK_ and I_SK_ activation must be reduced. We arrive at this hypothesis from an interpretation of experimental data in the literature.

If the P-type voltage-gated Ca^2+^ current (I_CaP_) is pharmacologically blocked in a full Purkinje cell morphology, its inherent trimodal (or tonic) firing pattern can be eventually replaced with a phase of bursting and then a depolarisation block [1], [2]. The waveform of this bursting is without the stereotypical change in firing rate/spike height, upon burst progression, that characterises dendritically generated bursting [3]. It is reported as similar to that observed in mechanically dissociated somas [3]. So, we hypothesise that this bursting is somatically driven and corresponds to the bursting observed in some isolated Purkinje somata [12]. Thus, we believe its study can be used to derive insight into the bursting of isolated somata bursting, and vice-versa.

We hypothesise that I_CaP_ block causes this outcome (bursting and then depolarisation block) because it equates to a combined I_BK_ and I_SK_ block. BK and SK channel activation is selectively coupled to I_CaP_ activation - experiments have shown that Ca^2+^ for activating BK and SK channels is provided *solely* by I_CaP_ flow in the Purkinje cell [2] i.e. BK and SK channels do not “read” the global intracellular Ca^2+^ concentration but singly the Ca^2+^ flux through I_CaP_. The concurrent reduction in BK and SK activity unlocks the somatic bursting state. Then, during this bursting mode, as the proportion of I_CaP_ molecules that are blocked increases over time, the BK and SK activity level falls further still. As SK activity falls, eventually SK cannot fulfill its role as the “burst terminator” and a burst cannot be terminated and a depolarisation block silence ensues. I_CaP_ block ensures a concurrent reduction in BK and SK activity: the individual block of BK or SK cannot switch a Purkinje cell out of the trimodal (or tonic) firing pattern [2], [4].

**Figure 5:**
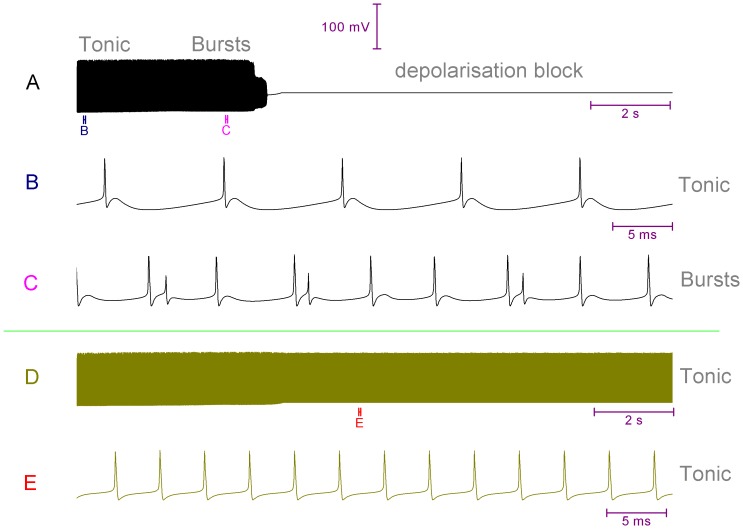
The bursting of the Purkinje soma model is gated by I_CaP_. *A*, Decreasing the I_CaP_ density to zero, with an arbitrary function of time (t) [density(t) = density(0) - (1*10^−7^)*t], shifts the somatic model activity from simple spiking, into bursting and then depolarisation block silence. *Panels B and C* correspond to the labelled parts of *Panel A* and highlight the transition from simple spiking to bursting. The bursting is irregular, with doublets interspersed with single spikes, as has been observed in some experimental recordings of somatic bursting [**12**]. *D*, I_CaP_ reduction can only unmask somatic bursting if the foundations for it are existent i.e. I_NaP_ and I_SK_ are both present at sufficient densities. If not, for example if the I_NaP_, density = 0 mS/cm^2^, then I_CaP_ reduction does not switch the somatic model out of tonic firing and the tonic firing state persists indefinitely. *Panel E* corresponds to the labelled part of *Panel D*. *All panels are scaled in the y axis (membrane potential) by the same scale bar, at the top of the figure (100 mV). Each panel has its own scale bar for the x axis (time)*.

*Figure 4F* shows the effect of decreasing the I_BK_ and I_SK_ densities to zero, with an arbitrary function of time (t) [density(t) = density(0) - (1*10^−5^)*t] that abstracts a progressing pharmacological block of BK and SK. It shifts the somatic model activity from simple spiking, into bursting and then depolarisation block silence. Figure 5 shows the effect of decreasing the I_CaP_ density to zero, with an arbitrary function of time (t) [density(t) = density(0) - (1*10^−7^)*t] that abstracts a progressing pharmacological block of I_CaP_. It shifts the somatic model activity from simple spiking, into bursting and then depolarisation block silence. The reduction in the I_CaP_ density causes this same switch in behaviour because it causes a concurrent decrease in I_BK_ and I_SK_ activity.

Note that I_CaP_ block (or I_BK_ and I_SK_ block) can only unmask somatic bursting if the foundations for it are existent: if I_NaP_ and I_SK_ (the burst initiator and terminator) are both present at sufficient densities. If not then I_CaP_ block (or I_BK_ and I_SK_ block) does not switch the soma model out of tonic firing and the tonic firing state persists indefinitely (Figure 5D & 5E).

### The Purkinje soma model can replicate Swensen and Bean's [12] elicited burst protocol

**Figure 6:**
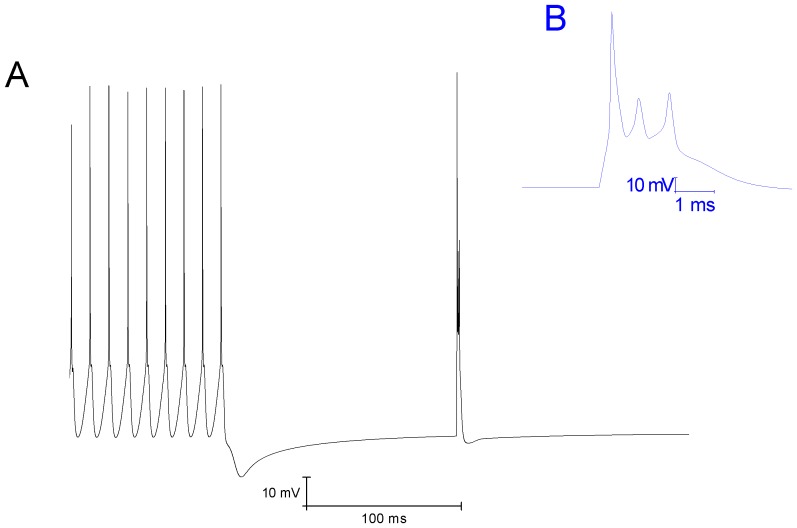
The Purkinje soma model can replicate Swensen and Bean's [12] elicited burst protocol. *A*, The model soma spontaneously fires simple spikes because it has an elevated I_BK_ and I_SK_ density that blocks bursting. At 100 ms it is driven to a hyperpolarised silence, and held there, by a maintained hyperpolarising current injection (−0.5 nA). This “held” cell is then driven to fire a single burst by a very short (1 ms) depolarising current injection (2 nA). Although the cell intrinsically fires simple spikes, it fires a burst in this protocol. So, the studied burst is elicited rather than spontaneous. *B*, The elicited burst at higher resolution.

For most of their study, Swensen and Bean [12] perform experiments upon an artificial form of somatic bursting. They take an isolated soma that spontaneously fires simple spikes and hold it at a hyperpolarised silence (−90 mV) with an enduring, hyperpolarising current injection. This “held” cell is then driven to fire a single burst by a very short (1 ms) depolarising current injection. Although the cell intrinsically generates simple spikes it fires a burst in this protocol. Hence the studied burst is elicited rather than spontaneous. This raises the concern that the mechanisms elucidated for the generation of such an elicited burst are not relevant for spontaneous bursts. Modelling can help address this issue. Our model's spontaneous bursting mechanism (I_NaP_ vs. I_SK_) primarily comes from an analysis of observations that Swensen and Bean [12] made for elicited bursts. Indeed, this mechanism allows the model to replicate their elicited burst results (*Figure 6*). But importantly, it also allows the model to fire bursts spontaneously. This demonstrates that elicited and spontaneous bursts are likely to have an equivalent basis, which vindicates the value of experiments that employ the elicited burst protocol.

## Discussion

An isolated Purkinje cell soma is a severely reduced system, but it is used in experimental research [11], [12]. In this paper we propose a biophysical basis to its bursting mode; Persistent Na^+^ current (I_NaP_) is the burst initiator and SK K^+^ current (I_SK_) is the burst terminator. This bursting mode is “gated” by the BK and SK currents (in a redundancy), which are in turn “gated” by the P-type Ca^2+^ current.

Is this bursting mode an artefact of the isolated soma system, or does it relate to physiological processes? Well, this bursting does not correspond to the bursting observed in the trimodal pattern of activity, in the full Purkinje cell morphology, because this bursting is dependent on the dendrites [3]. However, we suggest that the full Purkinje cell morphology can express the same bursting form as that observed in isolated Purkinje somata. This is, *in vitro*, upon the condition of P-type Ca^2+^ channels being pharmacologically blocked [1], [3]. So, this expression is itself in a severely artificial system: in cerebellar slices with the perfusion of a drug. There is presently no work that we know of that investigates or supports the hypothesis that, physiologically, Purkinje cells express a somatically driven bursting form and utilise it in information coding strategies. However, if they do employ this firing form, we can speculate that its bursting parameters might be under transmitter control as a computational feature. Transmitter control of I_SK_ has been described in many types of neurons [25], I_NaR_ may be regulated by phosphorylation [26] and I_NaP_ can be regulated by Nitric Oxide [27], Protein Kinase C [28] and external Ca^2+^ concentration [29].

In this paper, we have proposed the electrophysiological basis to bursting in an isolated Purkinje soma. We have shown how it could relate to different activity patterns that have been reported in full Purkinje cell morphologies [1], [3], and in isolated Purkinje somas [11], by other scientists in other experiments. This is the value of modelling – it can reconcile why different behaviour is observed in different experimental preparations and unify disparate behaviour into a single, cohesive framework.

In conclusion, we propose that the Purkinje cell has two separate and distinct modes of bursting: somatically generated and dendritically generated, which have dramatically different waveforms. We venture that the Purkinje cell may leverage this in its information coding strategies.
